# P-234. Clinical Factors Associated with Central Line Associated Blood Stream Infections (CLABSI) in Hospitalized Dialysis Patients

**DOI:** 10.1093/ofid/ofae631.438

**Published:** 2025-01-29

**Authors:** Jackson Morton, Anupama Neelakanta, Wendy Betts, Shelley Kester, Catherine Passaretti

**Affiliations:** Atrium Health, Charlotte, North Carolina; Atrium Health, Charlotte, North Carolina; Atrium Health, Charlotte, North Carolina; Atrium Health, Charlotte, North Carolina; Advocate Health, Charlotte, NC

## Abstract

**Background:**

We sought to understand factors associated with Central Line Associated Blood Stream Infections (CLABSI) in hospitalized patients receiving dialysis.

**Figure:**
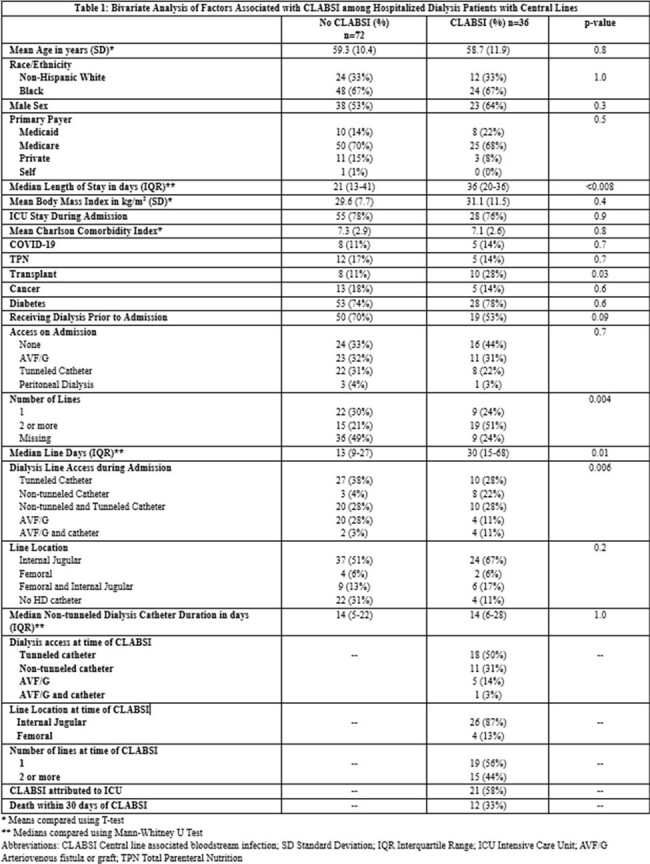

**Methods:**

7353 patients hospitalized between 2019 and 2021 with an ICD-10 code for dialysis and who had a central line accessed during their stay were identified. Demographics, comorbidities, central line characteristics, and length of stay data were collated from the electronic health record and linked to infection prevention CLABSI data. 38 cases on dialysis at the time of CLABSI were identified and matched to controls who did not have a CLABSI. Controls were matched to cases (2:1) by age (+/- 10 years), race, facility, admission date (+/-6 months) and intensive care unit admission during their stay. 2 cases were excluded due to inability to match.

Descriptive statistics were performed. Univariate and multivariable logistic regression models were used to assess for factors associated with increased odds of CLABSI.

**Figure:**
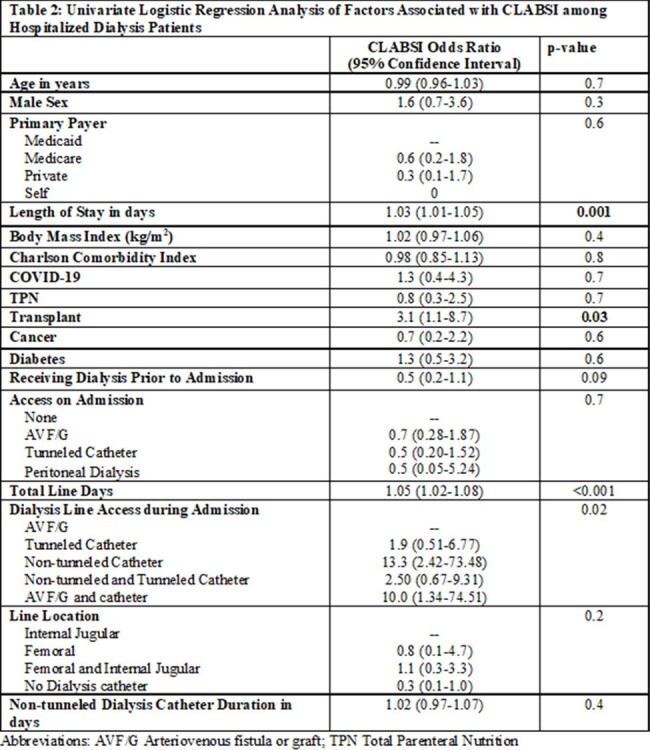

**Results:**

Dialysis patients with CLABSI were predominantly Black and male. 76% of cases were admitted to the ICU during their stay and 33% died within 30 days of CLABSI. Tunneled catheters, non-tunneled catheters and arteriovenous fistula/graft (AVF/G) were present in 50%, 31% and 14% of patients respectively at time of CLABSI. Cases were more likely than controls to have a non-tunneled catheter for hemodialysis access, longer length of stay, longer central line duration, and have had a transplant. (Table 1) Patients with non-tunneled catheters were 13 times more likely to have a CLABSI than those with AVF/G (p 0.02). Patients who received dialysis prior to admission had lower odds of CLABSI compared to acute dialysis patients (OR 0.5, p 0.08). (Table 2) After adjustment for age, sex, history of transplant, and being on dialysis prior to admission, increased length of stay (OR 1.03, p 0.01) and use of non-tunneled catheter for dialysis (OR 10.2, p 0.03) remained associated with increased odds of CLABSI. (Table 3)

**Figure:**
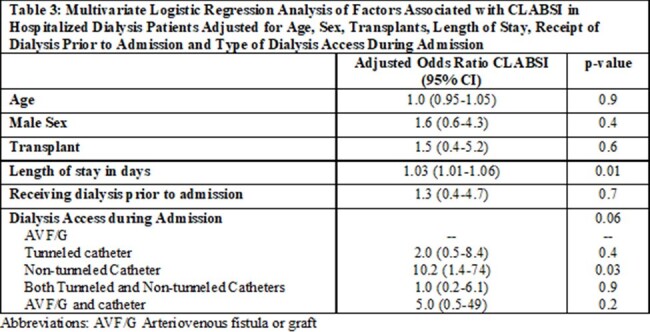

**Conclusion:**

The use of non-tunneled catheters and longer length of stay were identified as key predictors of CLABSI in patients on dialysis. These findings emphasize the need for strict infection control measures and careful monitoring of catheter types in this vulnerable population.

**Disclosures:**

**All Authors**: No reported disclosures

